# Beyond HIV-serodiscordance: Partnership communication dynamics that affect engagement in safer conception care

**DOI:** 10.1371/journal.pone.0183131

**Published:** 2017-09-07

**Authors:** Lynn T. Matthews, Bridget F. Burns, Francis Bajunirwe, Jerome Kabakyenga, Mwebesa Bwana, Courtney Ng, Jasmine Kastner, Annet Kembabazi, Naomi Sanyu, Adrine Kusasira, Jessica E. Haberer, David R. Bangsberg, Angela Kaida

**Affiliations:** 1 Center for Global Health, Massachusetts General Hospital, Boston, MA, United States of America; 2 Division of Infectious Disease, Massachusetts General Hospital, Boston, MA, United States of America; 3 Mbarara University of Science and Technology, Mbarara, Uganda; 4 Harvard T.H. Chan School of Public Health, Boston, MA, United States of America; 5 Research Institute McGill University Health Centre, Montreal, Canada; 6 Division of General Medicine, Massachusetts General Hospital, Boston, MA, United States of America; 7 OHSU-PSU School of Public Health, Portland, OR, United States of America; 8 Faculty of Health Sciences, Simon Fraser University, Burnaby, BC, Canada; International AIDS Vaccine Initiative, UNITED STATES

## Abstract

**Introduction:**

We explored acceptability and feasibility of safer conception methods among HIV-affected couples in Uganda.

**Methods:**

We recruited HIV-positive men and women on antiretroviral therapy (ART) (‘index’) from the Uganda Antiretroviral Rural Treatment Outcomes cohort who reported an HIV-negative or unknown-serostatus partner (‘partner’), HIV-serostatus disclosure to partner, and personal or partner desire for a child within two years. We conducted in-depth interviews with 40 individuals from 20 couples, using a narrative approach with tailored images to assess acceptability of five safer conception strategies: ART for the infected partner, pre-exposure prophylaxis (PrEP) for the uninfected partner, condomless sex timed to peak fertility, manual insemination, and male circumcision. Translated and transcribed data were analyzed using thematic analysis.

**Results:**

11/20 index participants were women, median age of 32.5 years, median of 2 living children, and 80% had HIV-RNA <400 copies/mL. Awareness of HIV prevention strategies beyond condoms and abstinence was limited and precluded opportunity to explore or validly assess acceptability or feasibility of safer conception methods. Four key partnership communication challenges emerged as primary barriers to engagement in safer conception care, including: (1) HIV-serostatus disclosure: Although disclosure was an inclusion criterion, partners commonly reported not knowing the index partner’s HIV status. Similarly, the partner’s HIV-serostatus, as reported by the index, was frequently inaccurate. (2) Childbearing intention: Many couples had divergent childbearing intentions and made incorrect assumptions about their partner’s desires. (3) HIV risk perception: Participants had disparate understandings of HIV transmission and disagreed on the acceptable level of HIV risk to meet reproductive goals. (4) Partnership commitment: Participants revealed significant discord in perceptions of partnership commitment. All four types of partnership miscommunication introduced constraints to autonomous reproductive decision-making, particularly for women. Such miscommunication was common, as only 2 of 20 partnerships in our sample were mutually-disclosed with agreement across all four communication themes.

**Conclusions:**

Enthusiasm for safer conception programming is growing. Our findings highlight the importance of addressing gendered partnership communication regarding HIV disclosure, reproductive goals, acceptable HIV risk, and commitment, alongside technical safer conception advice. Failing to consider partnership dynamics across these domains risks limiting reach, uptake, adherence to, and retention in safer conception programming.

## Introduction

Many men and women living with HIV and their partners want to have children [1–9]. As antiretroviral therapy (ART) becomes widely accessible, fertility desires and fertility of HIV-affected couples have increased, and are approaching rates observed among HIV unaffected couples [10, 11]. For those in HIV-serodiscordant partnerships, condomless sex to conceive introduces HIV transmission risk. While people living with HIV (PLWH), their partners, and providers are eager for safer conception programs [12–16], HIV prevention during periconception receives relatively little attention [17, 18] [14, 19–21].

Safer conception strategies include ART for the infected partner; delaying condomless sex until HIV-RNA suppression [22]; pre-exposure prophylaxis (PrEP) [23–29]; treatment for sexually transmitted infections [30, 31]; sperm washing (for male-positive couples); and male circumcision and home insemination (for female-positive couples) [17, 32, 33]. Formative research has focused on acceptability (to consumers [12, 16, 19, 28]) and awareness (of providers [15, 34–36]) of such methods. We conducted in-depth interviews in rural Uganda with PLWH and their uninfected or unknown-serostatus partners. The partner living with HIV reported that at least one member of the couple wanted a child in the next two years. Although our original research question focused on acceptability and feasibility of safer conception strategies, data revealed four domains of important partnership communication challenges to uptake of and engagement in safer conception services for HIV-affected couples.

## Methods

### Recruitment

Between 2005 and 2015, the Uganda Antiretroviral Rural Treatment Outcomes cohort (UARTO) recruited PLWH initiating ART in rural southwestern Uganda to determine predictors of ART adherence and virologic failure [37]. A Reproductive Health Component survey (2011–2015) assessed reproductive goals, and partnership dynamics, including partner’s HIV-serostatus and fertility desire [38]. Eligible index participants (“index”) for this sub-study were UARTO participants who completed the Reproductive Health Component, reported an HIV-negative or unknown-serostatus partner to whom they had disclosed their HIV status, and reported personal or partner pregnancy desire within two years. All study participants who completed an annual questionnaire between March 2013-April 2014 and who met the above criteria were approached by a research assistant to describe the linked qualitative study and determine interest in participating. After confirming interest and obtaining consent, index participants were asked to confirm HIV-serostatus disclosure to his/her partner, and then asked for permission for the study team to contact this partner. Partners (“partner” participants) were eligible for the sub-study after providing informed consent. Recruitment of index participants continued until data saturation was met.

### Data collection

Socio-demographic information about index participants was obtained from the annual UARTO questionnaire preceding the in-depth qualitative interview. Similar data were obtained from partners through a brief, interviewer-administered questionnaire.

Individual in-depth interviews (conducted separately with each member of the couple) explored reproductive goals, partnership communication, concerns about periconception HIV transmission risks, and feasibility and acceptability of five safer conception strategies: ART for the infected partner, PrEP for the uninfected partner, timing of condomless sex to peak fertility, manual insemination, and male circumcision.

Prior to asking questions about awareness and acceptability, interviewers described safer conception strategies using case-based narratives and images (S1 Fig) [39]. Two co-authors (AK, FB) drafted the vignettes inspired by previous qualitative research findings [34] and clinical experience working with HIV-affected couples in Mbarara. The vignettes were then shared with five members of the Mbarara clinical and research team who evaluated the clarity, face validity, language, and appropriateness of the vignettes, including whether they reflected familiar situations and created a story to which participants would relate. Several revisions were suggested and incorporated through an iterative process to arrive at the final vignettes. In parallel, three co-authors (LTM, AK, JK, AK) worked with members of the research team and an artist to draft the images. Initial drafts were reviewed, discussed, and revised by the Mbarara clinical and research teams and members of the study site community advisory board over several weeks. The images were assessed for clarity of message and representativeness of the characters, including whether the images reflected familiar situations.

Interviews were conducted in the local language by a qualitative research assistant and audio-recorded. Each member of the couple was interviewed separately, rather than as a couple, in order to clearly assess the views of both the index and partner participant. We were specifically interested in understanding each individual’s considerations as he/she imagined making decisions about safer conception. While conducting couple interviews could have offered additional insights into safer conception decision-making, such an approach was beyond the scope of this study [40, 41].

### Data analysis

Audio-recordings were transcribed and translated into English. Transcripts were coded independently by three researchers and analyzed using an iteratively developed codebook to explore emergent themes. Key data points were summarized, discussed, and compared for consistency and discrepancies to facilitate identification of connections among research questions, coding categories, and raw data. After the initial coding, discrepancies in HIV serostatus-disclosure, childbearing intentions, HIV risk acceptability, and understandings of the partnership commitment emerged from the data as barriers to choosing safer conception methods. These emergent findings were discussed with the full research and clinical team and identified as critical to informing the development of safer conception services for HIV-affected individuals and couples. Four members of the research team (including 2 of the primary coders) reviewed the transcripts and coding to classify the discrepancies across the 40 interviews with 20 dyads, select representative quotes, and summarizing the stories of two representative dyads. NVIVO software was used to organize the analysis. Quantitative socio-demographic data from the questionnaires are described using standard descriptive statistics.

### Ethics statement

Ethics approvals were obtained from Mbarara University of Science and Technology (Uganda), Partners Healthcare (Boston, USA), and Simon Fraser University (Burnaby, Canada). Approvals were also obtained from the Uganda National Council for Science and Technology and the President’s Office. Participants provided written voluntary informed consent. Consistent with standard research participation reimbursements at this site, participants were given 1 bar of soap, 1kg of rice, or 1 kg of sugar for their participation and reimbursed for any associated transportation costs.

## Results

### Participant characteristics

We conducted in-depth interviews with 22 indexes and 20 partners. Data from two index participants whose partners were not interviewed are excluded. The 20 included index participants (Table 1) had a median age of 32.5 years and a median of two living children; 55% were women, and 85% reported an HIV-negative partner while 15% reported not knowing their partner’s HIV-serostatus. 75% reported personal desire for a child, and 85% reported that their partner desired a child. Indexes were on ART for a median of 2.8 years. Of those with viral load results, 80% (16/19) had HIV-RNA <400 copies/mL at the UARTO visit preceding the in-depth interview.

**Table 1:** Socio-demographic data from index and partner participants.

	Index participants, living with HIV and receiving ART (N = 20) Median [IQR] or N (%)	Partner participants (N = 20) Median [IQR] or N (%)
**Female**	11 (55%)	9 (45%)
**Age (years)**	32.5 [IQR: 28–36]	34 (24–49)
**Personal HIV-serostatus**		
**Positive**	20 (100%)	7 (35%)
**Negative**	0	13 (65%)
**Unknown**	0	0
**Report of partner HIV-serostatus**		
**Positive**	0	17 (85%)
**Negative**	17 (85%)	0
**Unknown**	3 (15%)	3 (15%)
**Years on ART**	2.8 [IQR: 0.91–5.4]	Data not collected in questionnaire
**Childbearing desire (self)**		
**Yes**	15 (75%)	
**No**	4 (20%)	
**Don’t know**	1 (5%)	
**Perceived childbearing desire of partner**		
**Wants a baby with index**	17 (85%)	
**Does not want a baby with index**	2 (10%)	
**Don’t know**	1 (5%)	
**CD4 cell count (cells/mm**^**3**^)*	377 [IQR: 311–539]	
HIV RNA <400 copies at blood draw prior to IDI**	16 (80%)	
**Number of living children**	2 [IQR: 1–3]	3 [IQR 1–5]

* n = 19

** n = 3 HIV-RNA ≥ 400 copies/mL; n = 1 missing

Partners had a median age of 34 years. 65% reported an HIV-negative serostatus while 35% reported an HIV-positive serostatus. 15% reported not knowing the index participant’s HIV status.

### Overview of qualitative findings

Awareness of HIV prevention options other than condoms and abstinence was limited. All index partners were on effective antiretroviral treatment, but this was rarely identified as a strategy to decrease sexual transmission of HIV. Even with the narrative and image approach (S1 Fig) to explain safer conception methods, individuals’ awareness and understanding was insufficient for assessment of method acceptability or feasibility.

However, communication challenges within the dyad emerged as key barriers to uptake of and engagement in safer conception services. Partnership communication challenges occurred across four domains: (1) perception/knowledge of partner’s HIV-serostatus and disclosure, (2) childbearing intention, (3) HIV risk perception and acceptable level of HIV transmission risk, and (4) partnership commitment.

### Illustrative case studies

To illustrate these partnership communication domains, we first present case studies from two participating couples, using pseudonyms (**Table 2**). The first couple, “Esther” and “Edward”, revealed concordant reports of HIV-serostatus disclosure/knowledge, childbearing goals, HIV transmission concerns, and understanding of the nature and future of the relationship. Only two couples in our sample of 20 (10%) presented such concordance.

**Table 2:** Case studies (using pseudonyms) created from interviews with each member of the couple.

**Case 1: Esther and Edward. An example of a couple that is well-primed to engage in safer conception care**
Esther and Edward have been together for over two years. Esther is HIV-positive and on antiretroviral therapy (index), while Edward is HIV-negative (partner).
*Disclosure of HIV-serostatus*: Esther disclosed her HIV-serostatus to Edward at the beginning of their relationship. She knows that Edward is HIV-negative and he undergoes regular HIV testing to confirm his HIV-negative status. Edward is confused by the fact that he is still HIV-negative despite being with Esther for several years. He explains their HIV-serodiscordant status as being due to his “strong blood” and her adherence to ART.
*Childbearing intentions*: Esther and Edward have discussed their childbearing intentions and are in agreement regarding wanting one more child. When they discuss a potential pregnancy, they are concerned about HIV transmission risk to the baby, Esther’s health, and the financial demands of having another child.
*HIV risk perception and concern*: Esther and Edward have discussed a mutual desire to take precautions to minimize HIV-related risks before and during the pregnancy. They are aware of the prevention benefits of ART but do not know about any other ways to reduce sexual HIV transmission risk during pregnancy attempts. They are both eager to learn more about safer conception strategies.
*Partnership commitment*: Esther has 3 children from a prior relationship and 1 child with Edward. Edward considers all four children as his own. Both report having only one sexual partner, each other, and describe their relationship as emotionally supportive and “without quarrels.”
**Case 2: Janet and Julius. An example of a couple who illustrate communication discrepancies which may impede engagement in safer conception programming**
Janet and Julius have been together for two years. Janet is HIV-positive and on antiretroviral therapy (index).
*Disclosure of HIV-serostatus*: Janet and Julius share divergent stories about HIV disclosure and Julius’ HIV status. Janet was scared to tell Julius her HIV status even though he asked several times at the beginning of their relationship. Eventually, she decided to tell him as she worried that he would find out from someone else. Julius confirms that he knows Janet’s HIV-positive status. Janet reports not knowing Julius’ HIV status whereas Julius reports that he is also HIV-positive and has disclosed this information to Janet.
*Childbearing intentions*: Janet and Julius have not discussed their childbearing intentions. She has one child with a previous partner but doesn’t know whether Julius has any children. When she found out that she had HIV, she didn’t want to have another child, but she has since started to change her mind. She’s not looking to become pregnant but wouldn’t mind if it happened. Julius has 5 children with two previous partners. He doesn’t want more children because of financial constraints, but he suspects Janet does.
*HIV risk perception and concern*: Janet and Julius have not discussed a need to take precautions to minimize HIV-related risks before and during the pregnancy. Janet is not worried about transmitting HIV to Julius as she is on ART and trusts the related prevention benefits. Julius is also not concerned about HIV acquisition or implementing HIV prevention strategies, but this is because he is already living with HIV. However, since Janet does not know Julius’ HIV status, they were eligible to participate in our study exploring safer conception options for HIV-serodiscordant couples.
*Partnership commitment*: Janet has one child with a previous partner but isn’t sure if Julius has children. Julius reports having five children with two previous partners. They are both suspicious about the presence of other partners, outside of their partnership. Julius confirms that he has two partners in addition to Janet. Neither partner is confident about the future and commitment of their partnership.

The second couple described, “Janet” and “Julius”, is more typical of our sample, with interviews revealing substantial discordance across the four communication domains. In our sample, three couples presented discrepancy across all four domains (15%) while the remaining couples (15/20, 75%) presented discrepancies across 1 to 3 of the domains.

### Partnership communication themes regarding childbearing in the context of HIV

Below we describe the four partnership communication themes that emerged from in-depth interviews with individual couple members, illustrated by participant quotes. Across themes, data reveal how gender norms influence HIV-affected couples’ communication and negotiation around childbearing.

#### Theme 1. HIV-serostatus disclosure/knowledge

Although self report of HIV disclosure to partner was an index partner inclusion criterion, many partners reported not knowing the index’s HIV-serostatus. Similarly, the partner’s HIV-serostatus as reported by the index was frequently inaccurate, and mutual disclosure was uncommon. Different understandings of partnership HIV status manifested as the index and partner recounted different stories of how they informed or were informed about the partner’s HIV status.

***Index*:** When I came to know [his status] is when he was telling me that we [should] have a child. [I said], ‘So to have a child, you see, for me, I am sick [HIV-positive], and you are ok [HIV-negative]…?’ [He said], ‘Can’t you see me having a healthy baby?’ [I said], ‘That doesn’t mean that if you are healthy and I am sick that we can’t have a baby who is not sick.’ … And he showed me the card [with his HIV-negative results]. *Woman*, *Couple 11*

***Partner*:** … she told me she was sick. I also told her that I was sick. *Male*, *Couple 11*

Reasons for observed discrepancies in disclosure were rooted in gendered differences in disclosure experiences and risks. Male indexes commonly reported disclosing HIV status indirectly, often after the relationship was established:

***Index*:** The drugs [ART], she came and found them in the locker. She found them there and she asked me ‘what are these drugs for’? I told her.

**Interviewer:** Before you married her, you had already started taking ARVs?

***Index*:** Yes … Me, I don’t usually fear people. … by the time she saw the drugs, the thing [condomless sex] was already done. *Male*, *Couple 3*

In contrast, female index partners, reported struggling with disclosure, due to fears of violence, being “chased” from their homes, a loss of economic security, and having limited options for keeping and caring for a child.

***Index*:** He kept quiet like for a week [after she disclosed her HIV-positive serostatus], we stayed in such a situation until I reached a time of producing our child, I told him, ‘Give me money, the medicine, I will not manage it.’ … He refused.–*Woman*, *Couple 14*

#### Theme 2. Childbearing intentions

Participants commonly held divergent childbearing intentions and made incorrect assumptions about their partner’s desires. At times, the discordance in perceived childbearing intentions manifested as coercion. For instance, in this couple, the male index desires a child and expressed frustration with his partner’s reluctance to become pregnant. His partner reports attempting to dissuade him for her own health (she reports undergoing treatment for endometrial cancer) as well as the health of their older children; however, he is insistent.

***Partner*:** He says ‘I want us to produce, I want another child such that they can be three.’ … I tell him that … I am not refusing to produce, I will produce, but let us first look after these ones and they grow a bit and then we [will] produce. … He says ‘You produce now.’ *Woman*, *Couple 7*

Across interviews, reports of direct communication within couples about childbearing intentions were rare, with narratives highlighting gendered expectations and assumptions about partner’s desires. The female index partner below is eager to have a child “for” her partner, whom she thinks is childless. He, however, has a child and is reluctant to have condomless sex to conceive and risk acquiring HIV:

***Index*:** He was telling me that he wants a baby, because… he doesn’t have one. He says, ‘I want a child, … I want one child… I see that … your health is not good, I wouldn’t want to [make you have many children]. [He wants the child] in one year. *Woman*, *Couple 9*

***Partner*:** I have one child but he is in Tanzania. […] The one child that I have is enough.

[….] When I saw that she was sick [HIV-positive] I decided to use a condom, since I saw that I could not chase her [end the relationship]. *Male*, *Couple 9*

#### Theme 3. HIV risk perceptions

Confusion about HIV transmission risk to partners and infants was common, including disbelief that HIV-serodiscordance within an on-going sexual partnership was possible:

***Index*:** … to find that a person is sick [HIV-positive], sleeps with someone who is not sick, and she doesn’t get sick, it also confuses me, how does it happen? …. You can be sick and you produce a healthy baby? …There is no way I can explain. *Male*, *Couple 3*

Within mutually disclosed partnerships, concerns about HIV transmission risk differed between individuals. Index participants expressed confidence that her/his partner was protected from HIV, either because of ART or through “God’s will” and “strong blood”. However, HIV-exposed but uninfected partners seldom shared this view. Women, in particular, feared acquiring HIV but felt they had few means to protect themselves:

***Partner*:** “Condoms, he refused. … When we came from the hospital that very day when we tested, I told him now you see, you are sick. Me, I am not sick. Now let us use … the condom. … He said, he doesn't use a condom because he doesn't transmit the HIV virus.” *Woman*, *Couple 7*

Conversely, for the following couple, the female index is anxious about protecting her partner from acquiring HIV, whereas he is not worried because he believes he is protected.

***Index***: Now I ask myself, how can you stay with someone, you have the disease and it doesn't catch him? It confuses me, but my heart tells me that one day he will get it. Then what? *Woman*, *Couple 12*

***Partner*:** How it happens [HIV-serodiscordance]? You may have blood that doesn’t easily catch the virus. *Male*, *Couple 12*

Disparate understandings of HIV transmission risk yielded disagreement regarding acceptable strategies to meet childbearing goals. For instance, HIV-positive men who desired children were reported to seek seroconcordant partners:

***Partner*:** He said that … they tested him, … and found him with the virus, he knew that I can't stay with him anymore. He decided to look for another [woman], that's what he told me. I … told him, ‘Alright let me go [away] and you get a good family … [T]hen later when they failed each other, he came back. *Woman*, *Couple 16*

Women, however, described resigning themselves to the consequences of childbearing decisions made by the HIV-infected partner.

***Partner*:** I told him that we should always have sexual intercourse using a condom; he swore and refused. … [H]e got the phone and called my mom that … I want to divorce.

**Interviewer:** So then have you ever had sex with a condom?

***Partner*:** He refused [to use a condom]. *Woman*, *Couple 3*

Narratives revealed that HIV-negative women face pressures to bear children and secure their economic future that are often more compelling and urgent than avoiding HIV. This male index succinctly summarizes the issue as he describes his partner’s motivation to have his child:

***Index*:** What worries her is not having HIV but having no property. *Male*, *Couple 10*

#### Theme 4. Relationship commitment

All index participants reported personal or partner desire to have a child, and all partners consented to participate and verified a relationship with the index. Even so, within partnerships, individuals reported different reasons for and commitment to the relationship and, accordingly, different reasons for wanting to have (or not have) children. These discrepancies in understandings of partnership commitment compromised individuals’ ability to evaluate risks they would be willing to take to have a child. This female index participant describes her partner as a friend with whom she has no interest in having children. He describes her as a potential marriage partner and is eager to have a child with her, regardless of her HIV status.

***Index*:** This one is my friend… I use him. … When he is around we are together. … When I tell him that, “I don’t want [to have your child]”, you see him feeling really bad. I say that, “even if you do want, for me I don’t want to have a baby. You go and look for those who will produce.” *Woman*, *Couple 11*

***Partner*:** I went to her home and introduced and if God wishes it, we shall marry. *Male*, Couple 11

## Discussion

In this study, we found limited awareness of HIV prevention strategies beyond condoms and abstinence, thus precluding analysis of participant acceptability or feasibility of the presented safer conception methods. Interviews revealed substantial and frequent discrepancies in perceptions and interpretations of partnership HIV-serostatus, childbearing intentions, acceptable HIV transmission risks, and relationship commitment. These areas of disagreement across the dyad made it challenging to interpret participant assessment of method preference. For instance, if a partner living with HIV considered certain HIV prevention methods as acceptable for her partner, but her partner subsequently reported that he was living with HIV, then the acceptability of the preferred method was less relevant. Or, for instance, if a partner living with HIV described his preferred safer conception method, but his partner did not want any (more) children, then a fulsome discussion of preferred methods seemed premature. The described partnership discrepancies are likely to present challenges to increasing linkage, uptake, and engagement of HIV affected individuals and couples in safer conception programming. The particular risks reported by women in terms of communicating serostatus, reproductive goals, and relationship security highlight the importance of centering gender-informed approaches within safer conception programming to maximize women’s reproductive autonomy and safety [42].

A large body of literature explores the complexities of sexual partnerships and how those relationships impact HIV risk reduction behaviours and inform interventions [43–47]. The safer conception literature has not routinely explored these complexities and often assumes that safer conception services will be offered to mutually-disclosed HIV-serodiscordant couples with concordant fertility desires and attitudes towards risk [15, 34, 48–54]. Our findings highlight the importance of considering the complexities of dyadic dynamics when developing or implementing safer conception programming.

Low rates of direct serostatus disclosure to sexual partners have been described, and qualitative studies document fears of abandonment, blame, and violence associated with disclosure [47, 55–57]. In our study, index partners were only eligible for this study if they reported having disclosed to and knowing the partner’s status, yet we identified numerous instances whereby disclosure was incomplete. There are several possible explanations for these findings, including that index partners may disclose using non-verbal clues (e.g., leaving medications and/or HIV test results in plain view, alluding to serostatus), whereas partners may not have deduced the index’s status from these clues [56]. There likely exists a social desirability bias to report serostatus disclosure to the partner [58]. And finally, there may have been motivation to participate in the study in order to receive transport refund and other benefits [59]. Collectively, our data highlight the complexity and poor reliability of self-report of disclosure and the importance of addressing supported disclosure within safer conception programs.

An analysis of reproductive goals among HIV-serodiscordant couples in the Partners PrEP study revealed that men’s reproductive goals and practices often dominate [60], concurring with other studies [5, 7, 8, 61, 62]. Our study corroborates this while also describing partnership discordance in reproductive goals, a lack of communication regarding such goals, and assumptions about a partner’s reproductive goals based on gendered social norms. These findings underscore the importance of and challenges to offering both individual and couples-based safer conception services.

We also observed discrepancies in the level of HIV risk that partners were willing to assume to conceive a child–in part due to differential understandings of transmission risk. Disbelief that serodiscordance is possible negatively affected appreciation of the potential of safer conception practices–i.e., HIV-serodiscordance is simply not believed and the uninfected individual avoids testing, assuming that transmission has already occurred. Others believe that remaining uninfected while a partner is infected is due to factors that do not require intervention (e.g., “strong blood”). Again, given prevailing gender norms, regardless of who is infected, the male partner yields substantial power in determining if, when, and how risk reduction practices will be deployed, highlighting the importance of engaging men within safer conception programming [43, 63–68].

Effective support for and decisions about disclosure, reproductive goals, and HIV-prevention methods depend on understanding relationship dynamics and trade-offs made to initiate, sustain, and nurture partnerships. Encouraging communication about relationship and childbearing goals and plans must be considered a foundational component of supporting couples to choose and use safer conception methods [42, 69–71].

The communication gaps across the four described domains highlight an important opportunity for couples-based HIV counseling and testing (CHCT) in safer conception programming [24]. CHCT facilitates mutual HIV-serostatus disclosure, discussion of fertility intention, uptake of contraceptives when pregnancy is not desired, reductions in HIV and STI incidence [61, 72–75] and thus presents a platform to discuss safer conception based on couples’ HIV status and childbearing desires. The Ugandan Ministry of Health includes couples-based HIV counselling and testing as a key strategy to improve HIV prevention and treatment efforts [76], although uptake remains low [77]. Interventions to improve CHCT services and promote CHCT uptake are urgently needed. Leveraging the desires of individuals and couples to have healthy children may be an effective strategy to promote CHCT such that more couples are able to benefit from this crucial service. We hypothesize that framing disclosure within a holistic approach that focuses on building healthy families may support couples to overcome barriers to disclosure, and testing of this hypothesis is ongoing [19, 68, 78–80].

These data highlighting the importance and challenge of offering safer conception care to HIV-affected individuals or couples who are not able or willing to present with their partner. To be inclusive, counseling programs must offer services to individuals who may need options they can implement independent of a partner. For instance, in a safer conception program in South Africa, 100% of male index clients brought their female partners to the clinic, but only 56% of women brought their male partners, highlighting the known challenges that women face engaging their partners in reproductive health programming [81]. Studies in South Africa further highlight the complex relationship power dynamics that underlie communication, trust, decisions and risk behaviors [43, 44, 47]. The work reported here also reveals a gendered differential in safer reproductive decision-making.

Implementation of safer conception methods is most feasible with concordance between members of a couple across the four themes identified here. However, safer conception services might be best implemented in a harm reduction paradigm [54] wherein all HIV-affected persons (independent of partner participation and serostatus disclosure) are supported to make informed decisions about meeting their reproductive goals and CHCT is encouraged [24]. Qualitative studies highlight the dilemma providers face when offering safer conception advice to clients who are not yet able to disclose [82]. Developing strategies for providers to support individuals to approach safe disclosure and CHCT remains a priority [83–85] [24] [82].

This study has several limitations. Data come from a small sample and are not meant to be generalized. However, interviewing occurred until we reached thematic saturation. As a qualitative study, findings should be interpreted as hypothesis-generating for future research, and need to be evaluated using quantitative methodologies with larger samples. Index participants were engaged in care, accessing ART, and reported HIV disclosure and are, thus, not representative of the larger community of HIV-affected couples needing safer conception services. Given persistent stigma towards childbearing among PLWH, social desirability bias may have affected the information shared in the interviews.

## Conclusion

Awareness of the need for and potential of safer conception programming is growing. Our findings highlight that such programming must be prepared to address complex experiences of communication across multiple partnership domains, in addition to offering technical advice regarding safer conception strategies if we are to maximize reach of and engagement in safer conception programming to all HIV-affected individuals and couples requiring such services.

## Supporting information

S1 Fig Images and narratives for five safer conception methods. The following images and narratives were used to share information with participants about the various safer conception strategies to reduce sexual transmission between HIV-serodiscordant couple while allowing for conception. Images accompanied each narrative. Images and narratives were developed in close consultation with the clinical and research teams based in Uganda. Questions embedded throughout the narratives to encourage discussion are removed from this supplemental information.
(DOCX)
