# Supplementary material for: Beyond HIV-serodiscordance: Partnership communication dynamics that affect engagement in safer conception care
Source: PLoS One. 2017 Sep 7;12(9):e0183131. doi: 10.1371/journal.pone.0183131 (PMC5589112; doi:10.1371/journal.pone.0183131)
Supplement: S1 Fig — The following images and narratives were used to share information with participants about the various safer conception strategies to reduce sexual transmission between HIV-serodiscordant couple while allowing for conception. Images accompanied each narrative. Images and narratives were developed in close consultation with the clinical and research teams based in Uganda. Questions embedded throughout the narratives to encourage discussion are removed from this supplemental information. (DOCX) [file pone.0183131.s001.docx]

**A. Images demonstrating methods**


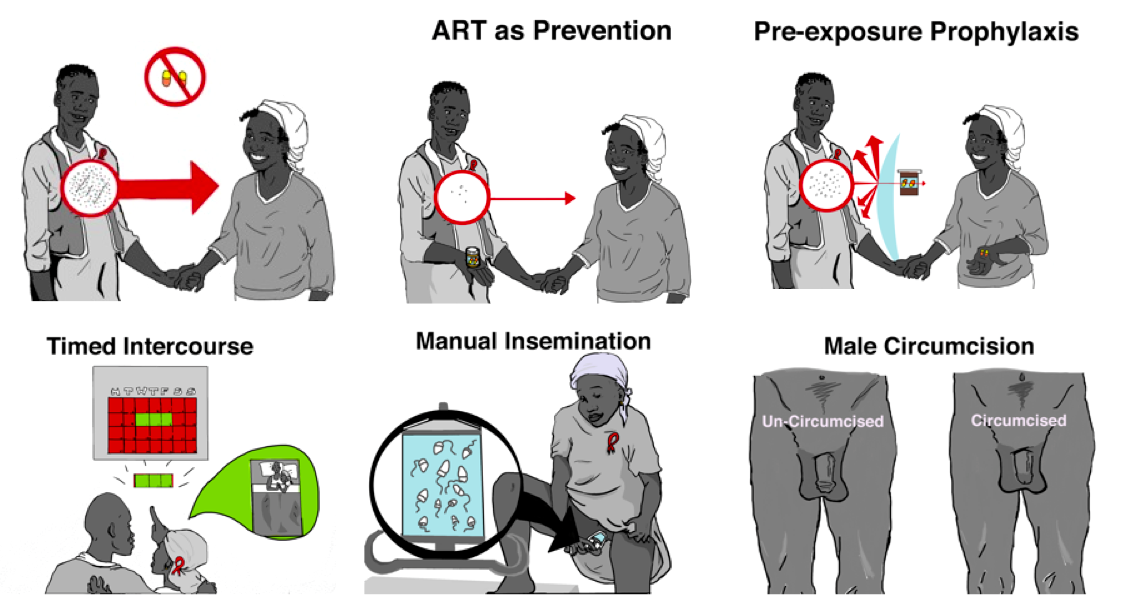


**B. Text read by interviewer**

Now, I’d like to tell you a story about a couple, named Fred and Maggie, who would like to have children. Maggie has HIV and Fred does not. In this story, Fred and Maggie learn about some of the specific strategies that can be used to lower the risk of HIV transmission to Fred, while still allowing for pregnancy.

After Fred and Maggie learn about a new strategy, I’ll ask you to tell me what you think and what you’ve heard about this strategy, if you know anybody who has used this strategy, and whether you have any questions or concerns about the strategy. Can we proceed?

Fred and Maggie are married. They live together in [local] town. Maggie is HIV-positive and Fred is HIV-negative. Maggie has been on antiretroviral therapy for the last two years. They have had two children together. The first one was a girl who was born HIV-positive and very sick. She died before her second birthday. The second one is a boy, who was born HIV-negative. He is now three years old. Fred and Maggie would like to have another child. Maggie knows that since she is on antiretroviral treatment, her risk of transmitting HIV to the baby is very low. But since they use condoms to prevent HIV transmission, they are not sure what to do to try to conceive a baby.

One day, Maggie’s auntie, Patience, comes by to say hello and Maggie shares with her that she would like to have another baby but does not want to give HIV to Fred. Patience is a village health worker who helps women with family planning. She thinks about Maggie’s worries for a while before reminding her that women are more likely to get pregnant during particular times in their menstrual cycle. And one way for couples to reduce the risk of transmitting HIV while trying to get pregnant is to use condoms most of the time. But when the woman is most fertile (based on counting the days since her last period), the couple can have sex without condoms to try to conceive. This method is known as “**timed intercourse**” and can be used to reduce the risk of HIV transmission from Maggie to Fred, while still allowing Maggie to become pregnant. This is the reverse of the “safe period” where couples *avoid* sex when the woman is most likely to get pregnant – as a form of birth control. ***(Refer to timed intercourse image. Questions and probes.)***

Fred and Maggie are curious about other methods that they might be able to use. Patience then remembers another method that a couple from her village used to become pregnant, called “**manual insemination**”. Patience tells them that the woman was HIV-positive and the man was HIV-negative, just like Maggie and Fred. In this case, to impregnate Maggie, Fred could ejaculate into a container and then use a syringe to insert his semen into Maggie’s vagina. Or they could have sex using a condom and then, after sex, reverse the condom to put the semen into Maggie’s vagina. Using this method of manual insemination, they could reduce the risk of HIV transmission from Maggie to Fred, while still allowing Maggie to become pregnant. ***(Refer to manual insemination image. Questions and probes.)***

Talking about these ways to reduce the risk of HIV transmission reminds Fred of a poster that he saw at the clinic promoting **male circumcision**. The poster encouraged men to come to the clinic to get circumcised to prevent HIV. Fred isn’t circumcised, so he wonders why the clinic is promoting circumcision. Patience tells him circumcised men have a lower risk of catching HIV during sex. Patience thinks that this could be another way to reduce the risk of Fred catching HIV from Maggie when they try to become pregnant. ***(Refer to circumcision image. Questions and probes.)***

Patience has to head back to her home and she thanks her hosts as she leaves. The next day, Maggie has an appointment at the HIV Clinic. Fred takes her to the clinic early in the morning. As they wait for Maggie to see the doctor, Fred sees his friend Wilson and his wife, Gloria, who is pregnant. After greeting each other, Wilson tells them that he started taking **antiretroviral treatment** about a year ago, when his CD4 count was low. He takes the medicines every day and now he has a very low level of the HIV virus in his blood. The doctor told them that because Wilson is on ARVs and has low levels of HIV virus, he is unlikely to transmit HIV to Gloria, who is HIV-negative, or to a baby. This information encouraged them to try to become pregnant. ***(Refer to treatment as prevention image. Questions and probes.)***

After Maggie’s clinic visit is done, she and Gloria go for a walk. Maggie tells Gloria that she would like to have another baby with Fred but they are nervous because of the risks involved. Gloria tells Maggie about a new study that she read in the newspaper about preventing HIV transmission. All of the people in the study were HIV-negative but had HIV-positive sexual partners, just like Fred and Gloria. In this study, the *HIV negative person took antiretrovirals*, to lower his/her risk of catching HIV. The article called this “**pre-exposure prophylaxis” or “PrEP**”. Doctors are still studying whether this method works and how PrEP might be used for couples who want to have a baby. ***(Refer to PrEP image. Questions and probes.)***

Maggie thanks Gloria for sharing this news with her. She meets Fred outside the clinic and together they head back home and discuss what they’ve learned. Fred and Maggie learned about five methods that couples can use to reduce the risk of HIV transmission while allowing for pregnancy. [Put cards out to show the 5 methods].
